# Effective treatment with afoxolaner (NexGard) of *Trixacarus caviae* in a pet guinea pig

**DOI:** 10.1002/vms3.70039

**Published:** 2024-09-06

**Authors:** Georgiana Deak, Miruna‐Maria Matei, Anca‐Alexandra Doboși, Aura Ursache, Andrada Negoescu, Marian Taulescu

**Affiliations:** ^1^ Department of Parasitology and Parasitic Diseases Faculty of Veterinary Medicine University of Agricultural Sciences and Veterinary Medicine of Cluj‐Napoca Cluj‐Napoca Romania; ^2^ New Companion Animals Veterinary Clinic Faculty of Veterinary Medicine University of Agricultural Sciences and Veterinary Medicine Cluj‐Napoca Cluj‐Napoca Romania; ^3^ Department of Genetics and Hereditary Diseases Faculty of Veterinary Medicine University of Agricultural Sciences and Veterinary Medicine Cluj‐Napoca Cluj‐Napoca Romania; ^4^ Dermatology Clinic Faculty of Veterinary Medicine University of Agricultural Sciences and Veterinary Medicine Cluj‐Napoca Cluj‐Napoca Romania; ^5^ Department of Pathology Faculty of Veterinary Medicine University of Agricultural Sciences and Veterinary Medicine Cluj‐Napoca Cluj‐Napoca Romania

**Keywords:** afoxolaner, guinea pig, isoxazolines, *Trixacarus caviae*

## Abstract

*Trixacarus caviae* is a sarcoptic mange mite infesting guinea pigs. Infestation in immunosuppressed animals produces severe dermatological problems, including alopecia, intense pruritus, hyperkeratosis and non‐dermatological issues (e.g., seizures). Treatment options are limited and include topical application of macrocyclic lactones or amitraz or injectable administration of ivermectin or doramectin. Considering the severity of the disease and the challenging treatment, the present paper aimed to determine the efficacy of oral afoxolaner in a severe case of infestation with *T. caviae* in a pet guinea pig. One female guinea pig was referred to the New Companion Animal Clinic due to severe dermatological problems. A clinical evaluation was done, and skin scrapings were collected and examined under the microscope. Small mites were detected and morphologically identified as *T. caviae*. The animal was treated with a single oral dose of 2.50 mg/kg afoxolaner, and the lesions, presence/absence of mites and intensity of pruritus were evaluated periodically until 2 months post‐treatment. A week after the medication, the lesions were milder, but pruritus was still present and was attributed to the healing process. Further examinations showed significant improvement with the complete remission of clinical signs and no mites at the microscopic examination after 4 weeks. Afoxolaner was safe and effective in this guinea pig for the treatment of *T. caviae* mange with no repetition needed.

## Graphical abstract

One female guinea pig was presented with dermatological problems and was confirmed to be infested by *Trixacarus caviae* mites. The animal was treated with a single oral dose of 2.50 mg/kg afoxolaner, with the complete remission of clinical signs and no mites at the microscopic examination after 4 weeks. Afoxolaner was safe and effective in this guinea pig for the treatment of *T. caviae*

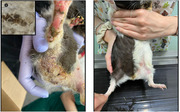

## 1 INTRODUCTION

The popularity of guinea pigs (*Cavia porcellus*) as pets has increased substantially over the past decade, meaning veterinarians will be more frequently dealing with their presence in their clinics. Most of the pathologies of these animals are caused or aggravated by poor maintenance and nutritional conditions (O'Rourke, 2004). Several ectoparasites are known to commonly infest guinea pigs including the sarcoptiform mites, *Trixacarus caviae*, (White et al., 2003). Sarcoptid mites dig burrows into the stratum corneum of the epidermis where females lay eggs. Sarcoptic mange is transmitted by direct and indirect contact (Jones et al., 2008). Infested animals can be asymptomatic, although the ones maintained in poor conditions or immunosuppressed can develop severe dermatological problems like alopecia, intense pruritus, scales, crusts, and hyperkeratosis and even non‐dermatological manifestations, like seizures (Beck et al., 2007). In addition, lesions can be aggravated by secondary bacterial infections (White et al., 2003). It should be noted that this parasite presents a zoonotic risk, as it can produce transient, itchy and papulo‐vesicular lesions in humans (Kummel et al., 1980). Diagnosis is based on the detection and morphological identification of mites using specific methods (White et al., 2016).

Treatment options are limited and mostly refer to off‐label administration of molecules applied on the skin or injectable. Bathing with amitraz (Mederle & Indre, 2009) and spraying fipronil (Malley, 1997) weekly are effective options, but in case of severe crusting, the absorption of molecules can be affected. Macrocyclic lactones like spot‐on selamectin were proven efficient on several occasions (Fisher et al., 2007; Honda et al., 2011), but repeated administrations are needed for complete remission. Injection with ivermectin has been described as effective but requires several administrations and the toxicity of ivermectin in guinea pigs must be considered (Ebel et al., 2023). Weekly doses of intramuscular 1% doramectin also proved effective after three administrations (Singh et al., 2013).

Isoxazolines are among the latest classes of ectoparasiticides described, which gained a lot of interest in the last few years. Currently, good efficacy has been proven for infestation with parasitic arthropods in dogs and cats (Zhou et al., 2022) and no resistance has been reported yet. Due to the lack of reported resistance to these molecules and the route of administration available (oral), isoxazolines are preferred options. Among these, fluralaner was successfully used in guinea pigs for the treatment of *Cimex lectularius* and *Rhipicephalus sanguineus* nymphs with 100% efficacy and no adverse reactions (Heckeroth et al., 2011). Afoxolaner was also used in snakes and rabbits with excellent results and no side effects (Alfonso Mendoza‐Roldan et al., 2023; Núñez et al., 2019; Sodelli et al., 2023).

Considering the severity of trixacaric mange in guinea pigs and the need for a better and easily administered form, the present study aimed to assess the efficacy of oral afoxolaner in a severe case of trixacaric mange in a pet guinea pig.

## 2 CASE PRESENTATION

A 3‐year‐old non‐spayed female guinea pig was presented in May 2023 to the clinic due to severe dermatological problems. Anamnesis revealed severe itching, pain signs, dermatological problems for about 1 year and different treatments (details unavailable) done at several clinics without long‐term success. The owner reported leaving the guinea pig outside in the garden for walks and using home‐produced hay. Clinical examination showed a normothermic patient (38.2°C) with a normal weight of 0.785 kg, manifesting restlessness and intense pain while handling it. The skin had multiple excoriations caused by scratching due to intense pruritus, and alopecia areas on the shoulders, back and hind legs.

**FIGURE 1:**
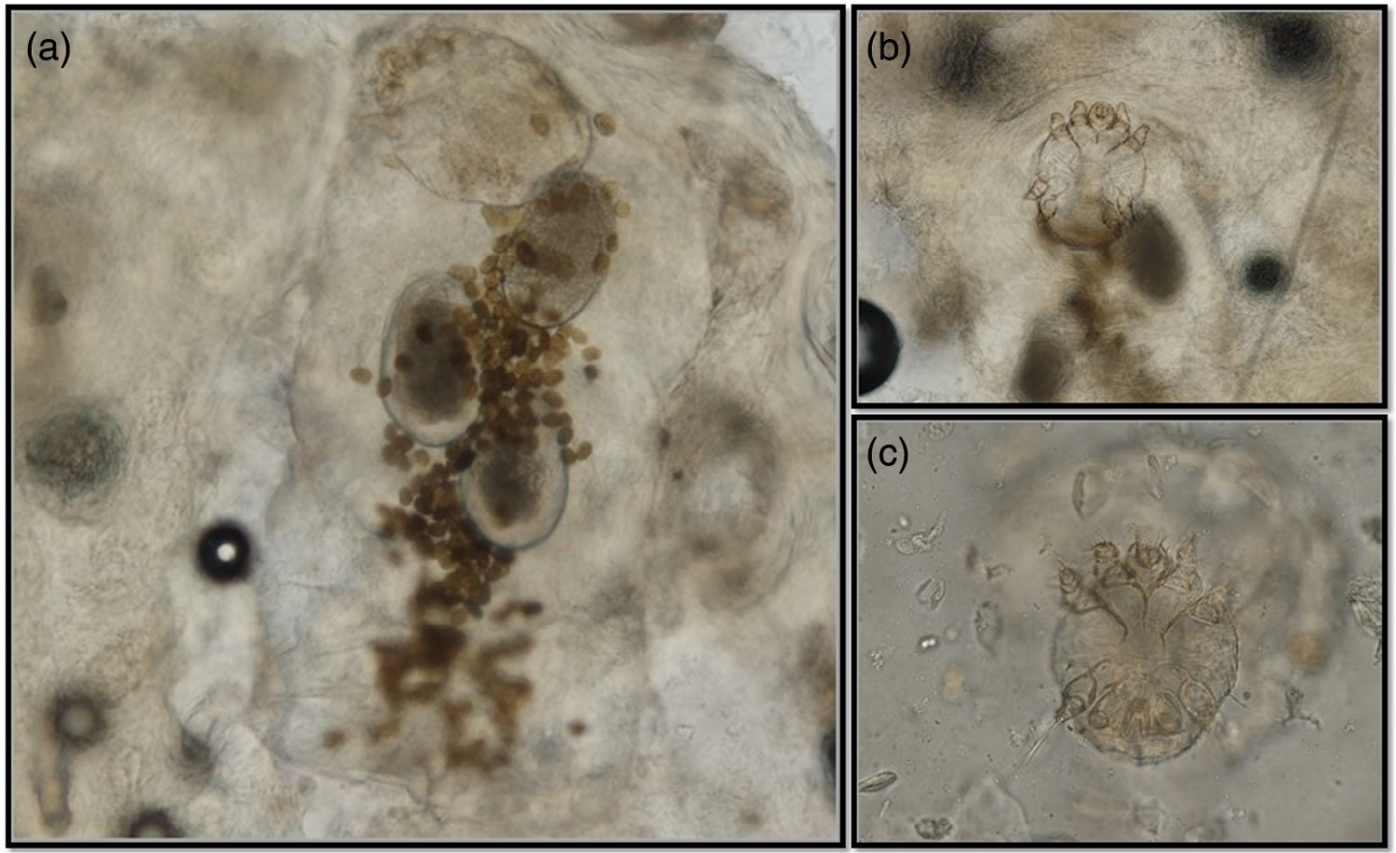
Microphotographs of different stages of *Trixacarus caviae*. (A) Eggs containing larva, faeces and one adult mite. (B) Ventral aspect of adult female mite. (C) Ventral aspect of adult male mite.

Skin scrapings were done from three different body areas and examined under the microscope in mineral oil. Different development stages of mites (more than 40 per slide) were detected (Figure 1A–C), morphologically consistent with *T*. *caviae* based on the small size, short legs and dorsal position of the anus in male mites, presence of simple setae and sharp spines on the dorsal part (Figure 1C).

A skin‐snip biopsy was also done to check for other secondary and/or associated changes, fixed in 10% formaldehyde for 24 h and further embedded in paraffin. Serial sections at 2‐µm thickness were done and routinely stained with hematoxylin and eosin.

**FIGURE 2:**
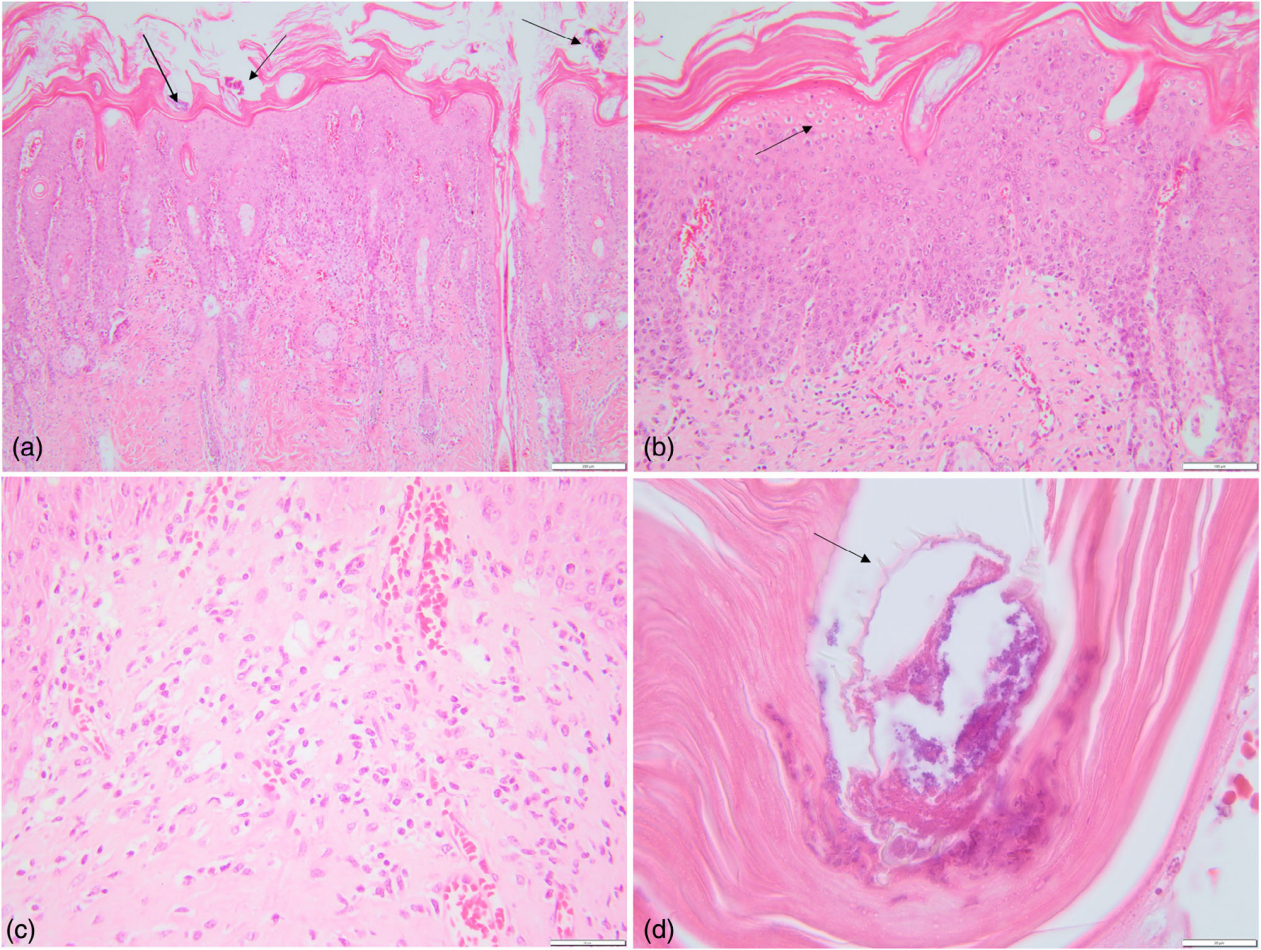
Photomicrographs of skin sections with chronic hyperplastic dermatitis with intracorneal *T. caviae* mites. (A) Multiple arthropod mites (black arrows) in the stratum corneum; (A and B) diffuse irregular hyperplasia of the epidermis and orthokeratotic hyperkeratosis; (B) mild spongiosis (black arrow). (C) Note the superficial dermis infiltrated with lymphocytes and rare plasma cells, macrophages and eosinophils. (D) Intracorneal arthropods show a chitinous exoskeleton, dorsal spines and fragments of skeletal muscle (black arrow). Hematoxylin and eosin (HE) stains.

Histology showed diffuse irregular epidermal hyperplasia with mild spongiosis and moderate diffuse orthokeratotic hyperkeratosis (Figure 2A,B). Numerous arthropod mites were detected in the stratum corneum (Figure 2A). In the superficial dermis, there was a perivascular to interstitial inflammatory infiltrate composed of a moderate number of lymphocytes and rare plasma cells, macrophages and eosinophils (Figure 2C). The arthropod mites were composed of a chitinous exoskeleton with dorsal spines, jointed appendages, striated skeletal muscle and digestive and genital tracts (Figure 2D). The histological diagnosis consists of chronic hyperplastic and hyperkeratotic dermatitis with intracorneal mites.

**FIGURE 3:**
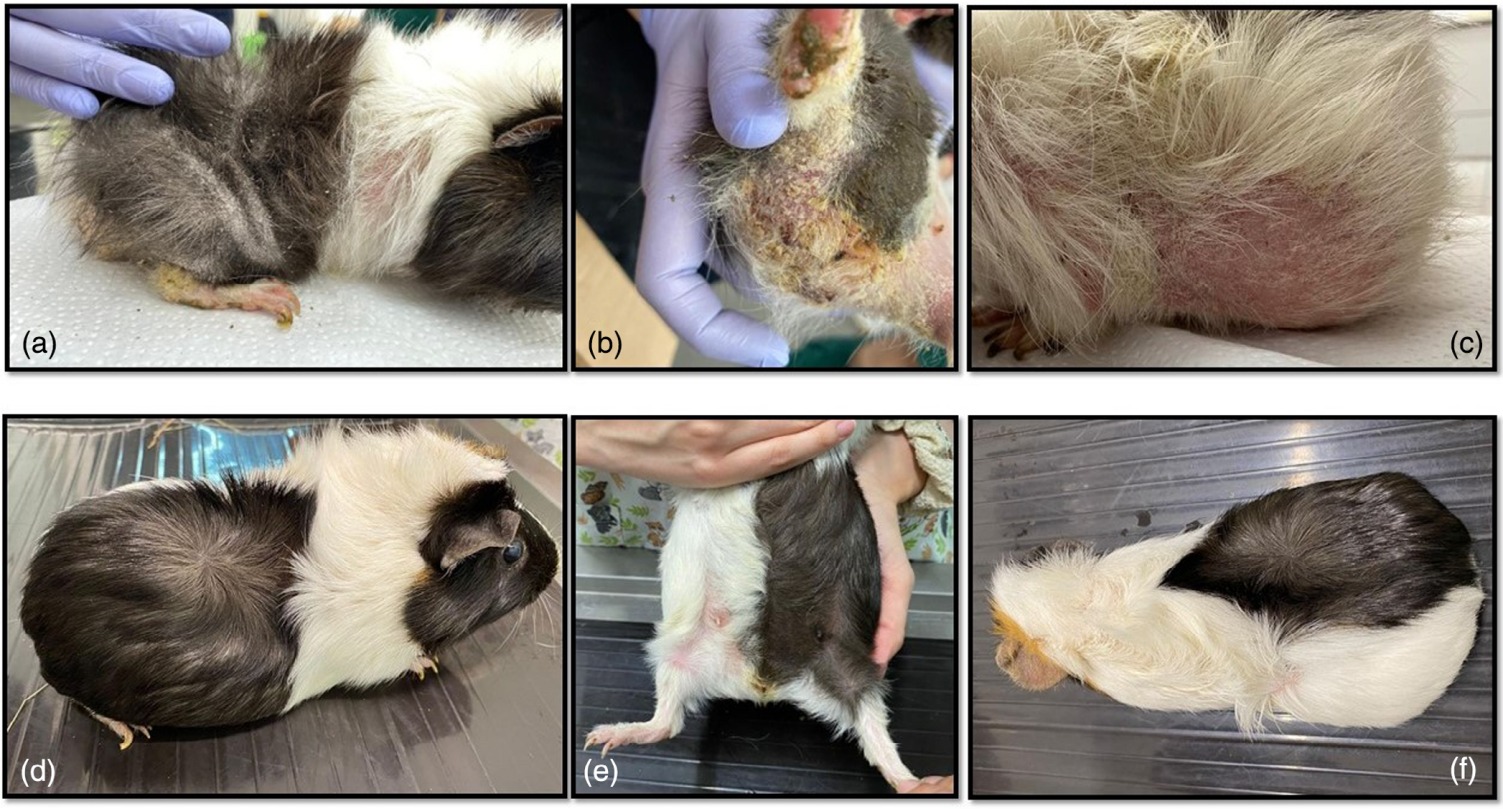
Clinical aspects of the infested guinea pig. (A) Day 0: lichenification, scales and crusts on the lateral part of the body; (B) Day 0: diffuse erythema, alopecia, scales and crusts in the inguinal area; (C) Day 0: diffuse alopecia and erythema on the lateral part of the body; (D–F) the last check‐up—full regrowth of the hair on the dorsal and ventral part of the body with no lesions.

Treatment was done with a single administration of oral 2.5 mg/kg afoxolaner (NexGard) (the smallest tablet for dogs 2–4 kg containing 11 mg was divided into 5.5 parts) mixed with EmerAid IC Herbivore (EmerAid LLC) in a syringe to facilitate the administration. For the off‐label treatment, written consent from the owner was obtained as well as an ethical permit from the Bioethical Department of the responsible facility (decision nr. 320 from 3 June 2022; available until 2025). Rigorous hygiene was implemented to protect the owners and to avoid reinfestations. Check‐ups were done every week for 1 month and then one more time after 2 months. One week after the treatment, the pruritus significantly reduced, and the lesions started to improve. Dead mites were found at skin scrapings (only 2–3 per slide). At 2 weeks post‐treatment, no mites were detected in none of the skin scrapings, but lesions were still present, and mild pruritus. At 3 weeks post‐treatment, there were no mites, no pruritus, the lesions resolved and hair started to grow back. At 1 and 2 months post‐treatment, the guinea pig was healthy with no signs at all of any dermatological problems and with the hair completely regrown (Figure 3D–F).

The owner was advised to avoid leaving the guinea pig for walks outside in the garden, to use commercial bedding and to consider prophylactic antiparasitic treatments for the future.

## 3 DISCUSSION AND CONCLUSIONS

The present case showed that a single oral dose of 2.5 mg/kg afoxolaner (NexGard) is safe and efficient for the treatment of *T*. *caviae* infestation in guinea pigs. The source of the infestation is not clear, as *T. caviae* is considered specific for guinea pigs and the animal was held in the same household since it was bought as pup. A theory could be that it was infested at the time of the acquisition but had a low mite burden and no clinical signs and maybe due to the development of immunosuppression with other causes, the clinical disease was triggered. The animal was left in the garden for daily walks, and the chances it acquired the infestation from there are unlikely as no wild hosts were described in the scientific literature for this mite. None of the people involved in handling the animal were affected. Although in the present case, environmental treatment was applied for the sake of safeness, afoxolaner was previously used in snakes infested by *Ophionyssus natricis* (Alfonso Mendoza‐Roldan et al., 2023) and in an African pygmy hedgehog infested with *Demodex canis* (Núñez et al., 2019) with no need for additional treatments, suggesting a good alternative for ectoparasitic infestations in many exotic animals, including guinea pigs, with an easy single administration. Besides these, afoxolaner proved efficient and safe also in a pot‐bellied pig infested with Sarcoptes *scabiei* with no adverse reactions observed and no neurological effects, suggesting the safety of use in pigs (Smith et al., 2020). In combination with milbemycin oxime (Nexgard Spectra), afoxolaner showed good efficacy after 1‐week post‐treatment in rabbits infested by *Psoroptes cuniculi* with no topical or environmental treatments (Núñez et al., 2020). In the present case, the use of prednisolone might have accelerated the healing process by contributing to the reduction of inflammation and pruritus. The administration of probiotics did not influence the efficacy of afoxolaner, but they might have contributed to the minimization of liver toxicity.

Currently, there are no specific products containing isoxazolines available for guinea pigs or other exotic animals; however, considering the current increasing interest in new companion animals and the proven safety and efficacy in various ectoparasites in these animal species, specific tested products would be beneficial. Afoxolaner has the advantage of single oral administrations that reduce the handling stress and do not influence the topical absorption when severe skin lesions are present like in the current case. As it is a flavoured chewable tablet, it can be easily divided for small animal administration, but sometimes efforts are necessary to convince the animals to consume it as its flavour was designed to be attractive for dogs, and handling stress during the examination can influence the food intake. Mixing it with the typical food seemed a good and easy option in the present case.

A single administration of afoxolaner (NexGard) at a dose of 2.50 mg/kg for the treatment of *T*. *caviae* infestation in a guinea pig was a safe and effective alternative. Further studies are needed for a more comprehensive evaluation of this molecule and its long‐term effects in rodents.

## AUTHOR CONTRIBUTIONS

Georgiana Deak and Miruna‐Maria Matei were responsible for the treatment of the animal and wrote the manuscript, Anca‐Alexandra Doboși revised the paper and assisted to the checkups, Aura Ursache described the lesions and Andrada Negoescu and Marian Taulescu performed the histological analysis.

## CONFLICT OF INTEREST STATEMENT

The authors declare no conflicts of interest.

## FUNDING INFORMATION

The authors declare that no funds, grants or other support were received during the preparation of this manuscript.

### ETHICS STATEMENT

Ethical permit was obtained from the Bioethical Department of the University of Agricultural Sciences and Veterinary Medicine of Cluj‐Napoca (decision nr. 320 from 3.06.2022; available until 2025).

### PEER REVIEW

The peer review history for this article is available at https://publons.com/publon/10.1002/vms3.70039.

## CONSENT STATEMENT

The owner gave their written informed consent for publication.

## Data Availability

The data that support the findings of this study are available from the corresponding author upon reasonable request.
